# Supplementary material for: Protective Role of Recombinant Human Thrombomodulin in Diabetes Mellitus
Source: Cells. 2021 Aug 29;10(9):2237. doi: 10.3390/cells10092237 (PMC8470378; doi:10.3390/cells10092237)
Supplement: Supplementary file 1 [file cells-10-02237-s001.zip › cells-1323926-supplementary.pdf]

Supplementary Materials

Protective Role of Recombinant Human Thrombomodulin in Diabetes Mellitus

Yuko Okano, Atsuro Takeshita, Taro Yasuma, Masaaki Toda, Kota Nishihama, Valeria Fridman D'Alessandro, Chisa Inoue, Corina N. D'Alessandro-Gabazza, Tetsu Kobayashi, Yutaka Yano, and Esteban C. Gabazza.

Table S1. Reagents used for flow cytometry analysis

Reagents	label	Isotype	Clone	source
anti-mouse Ly-6G/Ly-6C (Gr-1)	FITC	rat IgG2bκ	clone RB6-8C5	BioLegend, Inc. (San Diego, CA).
anti-mouse F4/80	PE	rat IgG2bκ	clone CIA3-1	BioLegend, Inc. (San Diego, CA).
anti-mouse CD11c	PE/Cy5	hamster IgG	clone N418	BioLegend, Inc. (San Diego, CA).
anti-mouse CD3ε	FITC	hamster IgG	clone 145-2C11	BioLegend, Inc. (San Diego, CA).
anti-mouse CD45R/B220	PE/Cy5	rat IgG2aκ	clone RA3-6B2	BioLegend, Inc. (San Diego, CA).
anti-mouse CD25	FITC	rat IgG1λ	clone PC61	BioLegend, Inc. (San Diego, CA).
anti-mouse CD8a	PE	rat IgG2aκ	clone 53-6.7	BioLegend, Inc. (San Diego, CA).
anti-mouse CD4	PE/Cy5	rat IgG2bκ	clone GK1.5	BioLegend, Inc. (San Diego, CA).
anti-mouse NK1.1	PE	mouse IgG2aκ	clone PK136	BioLegend, Inc. (San Diego, CA).
FasL	biotin	hamster IgG2	clone MFL3	BD Pharmingen (San Jose, CA)
FITC	streptavidin			BD Pharmingen (San Jose, CA)
FITC	annexin V			BD Pharmingen (San Jose, CA)

FITC, fluorescein isothiocyanate; PE, phycoerythrin; PE/Cy5, phycoerythrin/cyanine 5
